# What are Your Eyes Revealing? The Contemporary Bedside Neuro-Ophthalmological Examination

**DOI:** 10.21315/mjms2021.28.5.15

**Published:** 2021-10-26

**Authors:** Pei Meng Ng, Jafri Malin Abdullah, Zamzuri Idris, Abdul Rahman Izaini Ghani, Sanihah Abdul Halim

**Affiliations:** 1 Department of Neurosciences, School of Medical Sciences, Universiti Sains Malaysia, Kubang Kerian, Kelantan, Malaysia; 2 Department of Neurosciences, Hospital Universiti Sains Malaysia, Universiti Sains Malaysia, Kubang Kerian, Kelantan, Malaysia; 3 Brain and Behaviour Cluster, School of Medical Sciences, Universiti Sains Malaysia, Kubang Kerian, Kelantan, Malaysia; 4 Unit of Neurology, Department of Medicine, School of Medical Sciences, Universiti Sains Malaysia, Kubang Kerian, Kelantan, Malaysia; 5 Department of Neurosurgery, Sarawak General Hospital, Kuching, Sarawak, Malaysia

**Keywords:** bedside examination, eye, neuro-ophthalmological, smartphone

## Abstract

The neuro-ophthalmological evaluation is done to assess the integrity of cranial nerves II, III, IV, VI. This work does not intend to substitute the examination in a fully equipped ophthalmology suite. The aim of this manuscript is to describe bedside examination of the eye by using simple apparatus that are easily available. This work incorporates the usage of smartphone application, as smartphone is deemed an accessory in almost every resident’s pocket during review of patients as a consult in the emergency department. However, the essence of traditional physical examination remains the fundamental. Understanding of neuroanatomy and neurophysiology of the eye and the central nervous system can enhance the clinician’s performance at the bedside.

## Introduction

**Figure 1:**
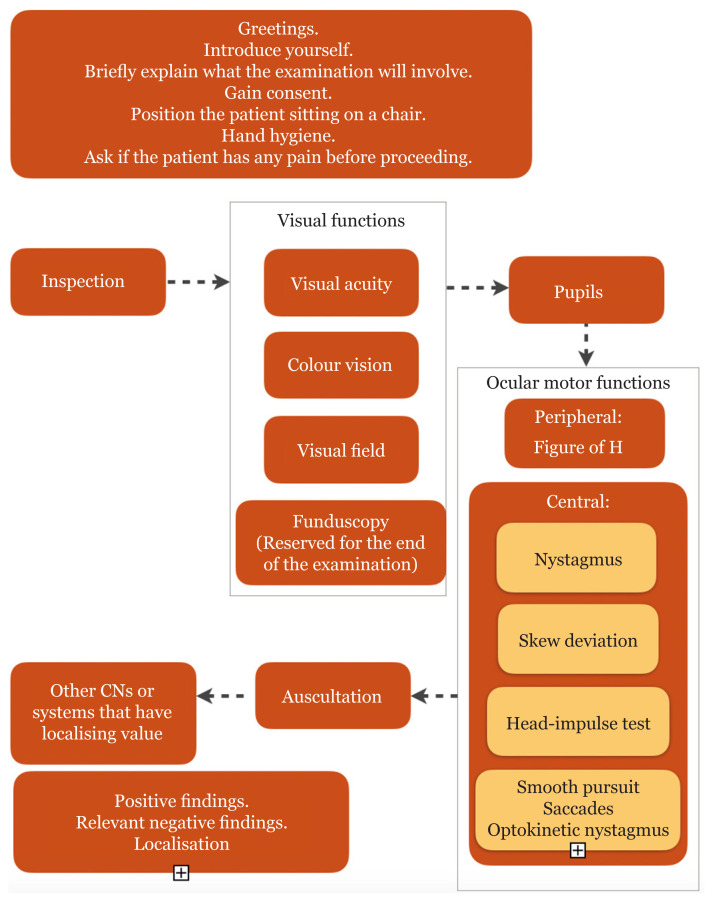
Flow of eye examination

**Figure 2:**
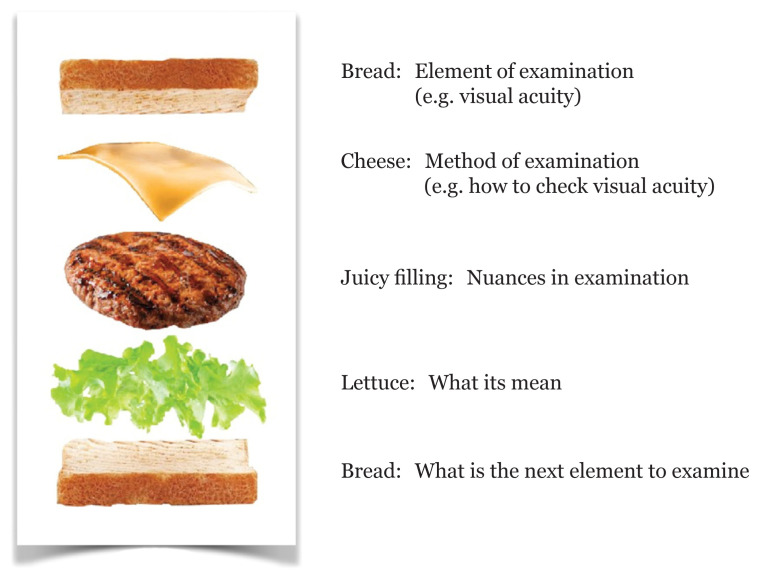
Intercalation ‘sandwich’ technique to describe each component of eye examination

The eyes are the window to the brain. Examination of eye is important to unravel the brain’s pathology. Besides clinical history, neurological examination remains a classical and an important bedside exercise. Bedside physical examination is a mixture of art and science. Examination of eye is a part that blends the fields of ophthalmology and neurology. For the examination of eye, the following five domains need to be addressed: i) inspection; ii) testing for visual function; iii) pupil evaluation; iv) ocular motor evaluation and v) auscultation (1–3). The flow of examination is crucial so that no steps will be missed. Figure 1 shows the flow and element of eye examination. In this article, an intercalation or ‘sandwich’ technique is used to describe each component of bedside neuro-ophthalmological exam (Figure 2).

## Inspection

To quote a phrase from Sir William Osler, “Don’t touch the patient – state first what you see, cultivate your powers of observation”. Therefore, the process begins with inspection. Generally, the process aims to search for any surgical scar, discharge or swelling over the orbital region, head posture and the position of eye in primary gaze. Then, a closer look is taken at the eyebrow, eyelid, conjunctiva and sclera. Next in inspection is to go behind the patient, looking down from above to check for proptosis, i.e. the Naffziger’s sign (4).

In the assessment of ptotic eyelid, since lower lid position may vary greatly, it is often helpful to measure the distance between the corneal light reflex and the upper lid margin. This number is referred to as the margin reflex distance (MRD1), where the normal value of MRD1 is between 4 mm–4.5 mm and MRD1 value is low in ptosis. MRD2 is the distance between the corneal light reflex and the centre of lower eyelid margin in primary gaze; however, MRD2 is not required in the evaluation of ptosis and a normal MRD2 value ranges between 5 mm–5.5 mm (5). The sole importance of adding MRD1 and MRD2 is that it provides palpebral aperture width. When there is a unilateral complete ptosis, the measurement of MRD can be skipped.

## Visual Function

### Visual Acuity

Testing visual acuity involves testing both distance vision and near vision. The patient is asked to put on spectacles if he normally does because the best corrected visual acuity is the acuity of neurologic significance (3). Proper indoor lighting is ensured because in dark surrounding, night vision will occur and this will decrease the visual acuity. Each eye is tested, whereby the first test preference is given to the better eye. The untested eye is occluded using palm, reason is to avoid view through the fingers and that no pressure is applied to the globe. Such pressure can temporarily cause refractive error and raise the intraocular pressure. The use of validated smartphone application as the bedside screening tool is proposed (6–8). The smartphone visual acuity is recorded at a distance as per the software instruction. In Malaysia, visual acuity is documented in meters. According to Medical Examination Standards for Vocational Driver’s Licensing in Malaysia 2011, the license maybe granted if visual acuity is 6/12 or better with or without correction glasses. A person with 6/12 vision means he has to be in 6 m to see what a normal individual can see at 12 m. For near vision, there is also a smartphone application available. In the supplementary video (https://youtu.be/Qop26igLg_g), the traditional near vision chart is employed, where the near vision chart is held 30 cm (the routine working distance) from the patient and the patient is then requested to read sections of print. Normal near vision is N6 (9).

### Colour Vision

Defect in red colour vision is very sensitive indicator of optic nerve disease. At the bedside, any red object, e.g. bottle cap or red colour tip of pen, can be used. The tip is viewed separately with each eye and the apparent colours are compared. Examiner can guide the patient to describe the red. With an abnormal optic nerve, the colour appears washed out, browner or less red. Correction of refractive error is necessary (1, 3).

### Visual Field

Bedside testing of visual field by using confrontation test by comparing the patient’s visual field with the examiner’s own visual field. Normally, there are two methods of confrontation tests. The first one is finger-counting fields. The examiner presents his hands on each of the two hemifields and the patient is asked whether both hands can be seen and whether any differences are noted in clarity or brightness. The examiner’s hands are then presented in two quadrants at a time, and again, the patient is asked to make comparison. Then, one, two or five fingers are presented in each quadrant and the patient is asked to report how many fingers are seen. The fingers can be presented for just a moment or for a longer period. When an area of depressed field is discovered, the examiner’s hand is moved from the depressed field to the normal field, where the patient is asked to notify when targets are seen clearly. Another method is outline perimetry. The examiner covers one of the patient’s eye and closes his own corresponding eye. The patient fixes his gaze on the examiner’s nose. Before that, patient is first informed to inform the examiner when a target is first seen. The target can be a wiggling finger that is brought in from periphery. The four quadrants of vision are tested separately. In Riddoch phenomenon, patient is able to detect movement in an area of field but not static target. In this consideration, finger-counting field is a better option. There are two nuances in confrontation test, the first one is a distance between patient’s eye and examiner’s eye of 50 cm (1), the second point is the wiggling finger oscillation of less than 5° (10). In normal condition, there should be no observation of any visual field defect. However, a unique patterned visual defect can help to localise the lesion along the optic pathway, for example, bitemporal hemianopia, which suggests lesion at the optic chiasm due to disruption of decussating nasal fibres (1). After visual field is examined, the blind spot is determined using a red pin. The red pin is moved from the point of fixation halfway between the examiner and the patient, laterally along the horizontal meridian until the examiner is able to find his own blind spot. Patient is then requested to inform examiner when the red pin disappears. Physiologic blind spot is located approximately 15° temporally in each eye. On the other hand, enlarged blind spot can be due to papilloedema.

## Pupil Evaluation

### Light Reflex

The room light is dimmed and the patient is informed that there will be flashes of light in his eyes. Next, a bright light is shone in one eye. The patient is ensured to be looking into the distance and not at the light. Subsequently, examiner is to observe for the reaction of that pupil (the direct light reflex). All of the above steps are repeated and the reaction in the other pupil is observed (the consensual light reflex) (1, 3). The normal pupil constricts promptly when light is shone on the ipsilateral retina. The contralateral pupil constricts because the fibres decussate at the optic chiasm and at the pretectal nucleus. In a diseased left optic nerve, the patient has equal pupils and no direct light reflex over the left eye but has a consensual light reflex on the left when the right retina is shone with light (1).

### Swinging Light Test

This test should be performed by alternately swinging the light from one eye to the other. The key is to keep it on the new eye for 3 sec–5 sec intervals. Nuances for performing swing light test is when moving the light beam between the eyes, using a U-shaped motion to avoid the pen torch crossing the visual axis, which may stimulate accommodation. Then, the examiner observes the pupillary response, as the light is shone into the eye (1, 3). In a healthy individual, the initial pupil response in each eye is constriction. In relative afferent pupillary defect (RAPD), the initial response when the light is swung to that eye is relatively dilated because the brain perceives less light (1).

### Accommodation Reflex

The examiner places his finger 10 cm in front of the patient’s nose. Patient is then asked to look into the distance and then at the examiner’s finger. The pupils’ constriction and eye convergence are observed for their reaction to accommodation (1, 3). Accommodation reflex comprises pupillary constriction, eye convergence and lens thickening. If the patient is blind, examiner may alternatively instruct him to look at his nose as his proprioception (sense of body parts) that is intact. This is to point that accommodation reflex does not require vision.

## Peripheral Ocular Motor Function Evaluation

### Figure of H

**Figure 3:**
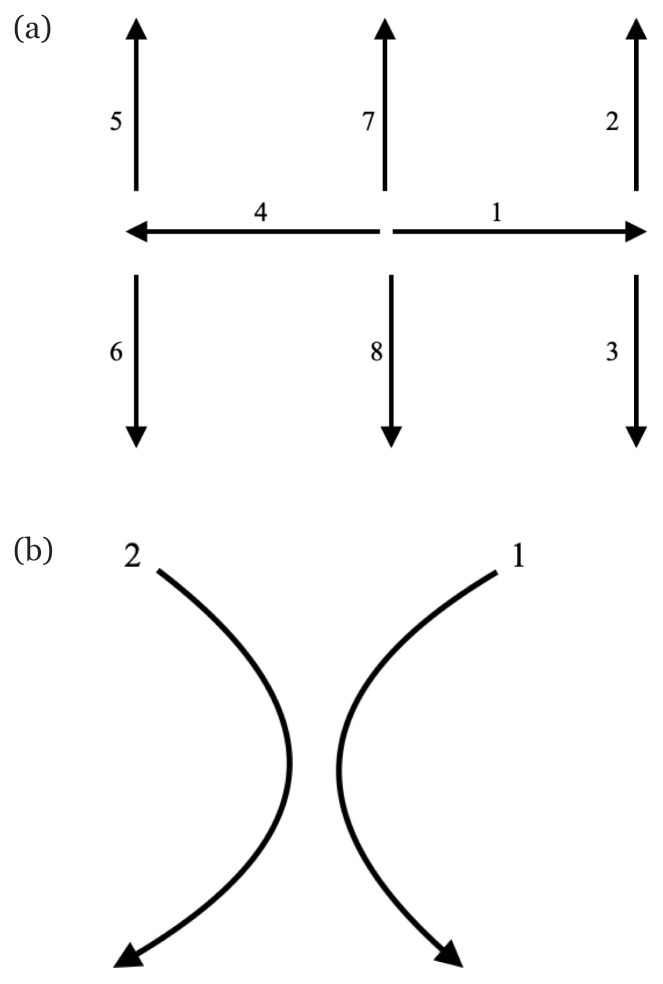
Direction of finger movement to examine extraocular muscle movement

Patient is asked to fixate on examiner’s finger. The finger is held up in the midline, about 50 cm away from the patient in the centre of his gaze. The examiner then requests patient to follow it with his eyes without moving his head and to inform the examiner if he sees double. The examiner can then hold the patient’s chin lightly to prevent head movement. The examiner moves his finger, as illustrated in Figure 3a. The examiner then observes for nystagmus and smooth pursuit.

All extra ocular muscles assessment can be done by having the patient follow the clinician’s finger in the shape of ‘H’ (1, 3). Another movement is added, where the figure ‘X’ is to isolate the superior oblique muscle movement, as in Figure 3b, by having the patient looking down and in albeit the function of superior oblique muscle is to move the eye down and out; however, this is difficult to ask the patient to look down and out, due to actions of lateral rectus and inferior rectus muscles. When patient looks down and in, the action of inferior rectus muscle is cancelled by contraction of the medial rectus muscle (11). Generally, majority can rotate their eyes readily, but the limits of rotation may vary between individuals. However, the most important point for diagnosing paralysis of any extraocular muscle is the differences between the two eyes during each plane of eye movement (3).

## Central Ocular Motor Function Evaluation

### Test for Skew

The patient is instructed to fixate at a point. Next, the examiner alternately covers one eye after the other. Compensatory vertical realignment of the eye that was just uncovered is observed. In normal condition, there is no vertical deviation of the eyes (2). Skew deviation occurs when there is a damage to the central otolithic pathway passing inside or near the medial longitudinal fasciculus.

### Head Impulse Test

The patient is asked to fixate on examiner’s nose. Head rotations are then applied side by side gently and a head thrust is performed with warning. It is important to apply head impulse test (HIT) with sufficient high peak velocities (120° s^−1^–150° s^−1^) in order to achieve an ipsilateral assessment of peripheral vestibular function (2). Normal HIT is when the eyes remain stable in space. On the other hand, for abnormal HIT there is delayed catch-up saccades (2).

### Test for Saccades

The test for saccades is to test fast saccadic eye movement by both horizontal and vertical planes (2). The examiner is to face the patient with both of the former’s hands out at about 30 cm apart from side to side and about 30 cm in front of the patient. Then, patient is asked to look from one hand to the other quickly. The eye movements are observed: Are they full? Do they move smoothly? Do they move accurately? Special attention is given when observing the speed of adduction. The process is then repeated with the examiner putting his hands vertically one above the other at about 30 cm apart and the patient is asked to look from one hand to the other. When the patient aims for a visual target quickly, normally, the saccade can be executed accurately. If there is eye undershoot, it is called hypometria; whereas an overshoot is called hypermetria. Lesion in the dorsal vermis and fastigial nucleus leads to saccadic dysmetria (12).

### Optokinetic Nystagmus

Optokinetic nystagmus (OKN) can be triggered by a moving visual pattern. It is traditionally tested by cloth tape or a handheld rotating drum (2). It is proposed that an OKN stripe is employed in smartphone application. The speed of the rotating drum increases by clicking on the screen. By turning the smartphone direction, the rotating drum direction can be changed. The patient is requested to look at the screen and count how many bars pass the display centre. The examiner observes for symmetrical OKN response. Normal OKN response is a slow involuntary pursuit movement iteratively, followed by a quick saccadic movement in the opposite direction in order to fixate the next new target that is entering the visual field. In patient with unilateral parietal lobe lesion (i.e. right), the slow pursuit phase of the OKN may be absent when the stimulus is moving toward the side of the lesion (i.e. right). The loss of the pursuit phase is due to interruption of efferent pathways from the parietal cortex to the conjugate gaze centre in brainstem (13).

## Funduscopy (Visual Function)

This is a part of assessment of visual function, where it reserved to the end of examination. Direct ophthalmoscope is small, light and inexpensive. Patient cooperation is another important factor. The room is darkened and the patient is asked to look at a distant object. For examination of right retina, the examiner holds the ophthalmoscope in the right hand and uses his right eye. Error of refraction in the patient or examiner eyes can be corrected by the use of lenses in the ophthalmoscope. The examination is begun by finding the optic disc, then the macula, and the surrounding macula (14). Papilloedema is caused by increased intracranial pressure. Fundus findings include the blurring of optic disc marking with obliteration of physiological cup, hyperaemic disc, dilated retinal vessels, haemorrhages, et cetera. In the late stage, optic atrophy will set in (9).

## Auscultation

A French physician, Laennec, had discovered auscultation and he was the creator for stethoscope (15). Auscultation is best done in a quiet place with a relaxed patient and examiner. When listening for an orbital bruit, the bell of the stethoscope is used to auscultate the patient’s closed eye. The patient should be instructed to open both eyes and gaze at a point across the room in order to eliminate the noise of rhythmic eyelid flutter. Then, the eyelid can be passively shut using the bell of the stethoscope (16). At the end of auscultation, the patient is instructed to hold his breath. Orbital bruit is usually faint and of high pitch. Detection of a bruit over the eyeball can be due to local pathology, e.g. carotid-cavernous fistula; systemic disease, e.g. anaemia or irradiation from distant structure, e.g. aortic stenosis (17).

## Conclusion

The correct sandwich is balance and tasty, it needs to be taken as a whole to taste great. As with bedside eye examination, the layered organised approach can provide the patient a feel of professionalism besides helping clinician to localise the lesion and to decide for subsequent ancillary study. It has been found that smartphone application can be a convenient aid in bedside neurologic examination, where further validation is required. This work ends with the words from Sir William Osler, “Teach the eye to see, the fingers to feel, and the ear to hear”. Practising and focusing on nuances in each step will reflect a more elegant, polished bedside neurological examination.
